# DOA Estimation and Self-calibration under Unknown Mutual Coupling

**DOI:** 10.3390/s19040978

**Published:** 2019-02-25

**Authors:** Dong Qi, Min Tang, Shiwen Chen, Zhixin Liu, Yongjun Zhao

**Affiliations:** National Digital Switching System Engineering and Technological Research Center (NDSC), Zhengzhou 86-450001, China; tangminmvp@126.com (M.T.); ndsccsw@126.com (S.C.); liuzhixin54@sina.com (Z.L.); zhaoyjzz@163.com (Y.Z.)

**Keywords:** DOA estimation, direction-dependent mutual coupling, time-frequency distribution, self-calibration

## Abstract

In practical applications, the assumption of omnidirectional elements is not effective in general, which leads to the direction-dependent mutual coupling (MC). Under this condition, the performance of traditional calibration algorithms suffers. This paper proposes a new self-calibration method based on the time-frequency distributions (TFDs) in the presence of direction-dependent MC. Firstly, the time-frequency (TF) transformation is used to calculate the space-time-frequency distributions (STFDs) matrix of received signals. After that, the estimated steering vector and corresponding noise subspace are estimated by the steps of noise removing, single-source TF points extracting and clustering. Then according to the transformation relationship between the MC coefficients, steering vector and MC matrix, we deduce a set of linear equations. Finally, with two-step alternating iteration, the equations are solved by least square method in order to estimate DOA and MC coefficients. Simulations results show that the proposed algorithm can achieve direction-dependent MC self-calibration and outperforms the existing algorithms.

## 1. Introduction

DOA estimation, as an important branch of array signal processing, is widely used in radar, sonar, radio astronomy and other fields [1]. In the past decades, many classical algorithms have been proposed which perform well in ideal situations, such as MUSIC, ESPRIT and other subspace-based algorithms. However, in practical applications, the performance of above algorithms suffers due to the effect of gain/phase uncertainties [2], sensor position perturbation [3] and mutual coupling (MC) [4], among which the mutual coupling caused by mutual excitation of array elements are common in engineering and makes the estimation accuracy deteriorate seriously. 

A number of methods have been proposed for DOA estimation in the presence of mutual coupling, which can be classified into two types: active-calibration [5] and self-calibration [6]. The active-calibration method which makes use of the calibration sources whose DOAs are exactly known, can achieve high DOA accuracy with low computation complexity. However, the existence of the calibration sources increases the additional cost of the system, and the performance of the algorithm deteriorates rapidly in the presence of DOA errors of the calibration sources, which is inevitable in practice.

On the contrary, self-calibration method is preferable since it does not require any prior knowledge of source locations and accomplishes the DOA estimation and error calibration online. In [7], an iterative algorithm is proposed for the estimation of DOA and MC coefficients, however, the result will converge to the local optimum if the initial values deviate far from the real ones. For uniform circular arrays, a self-calibration method is proposed based on rank-reduction estimator by using the complex symmetric Toeplitz property in [8]. The algorithm only needs one-dimensional search which lowers the computational complexity, but its parameter estimation is prone to be ambiguous. Moreover, reference [9] and [10] utilize alternating iteration and recursive estimation to solve this problem, respectively. Recently the methods which make use of instrumental sensors for array calibration has also been developed [11,12]. They exploit the fact that only part of the new array has mutual coupling or other errors after adding instrumental sensors into the original array. But in practice, it is impossible to obtain the ideal instrumental sensors.

The above algorithms are only adopted to direction-independent mutual coupling which is modelled with a single matrix. As it is established under the assumption of the omnidirectional antenna array, the model becomes ineffective when the array elements are not omnidirectional antennas. However, in practical engineering, due to the limitations of the manufacture and the working environment of the antenna, the array elements have directional beam pattern in general [13]. As a result, the mutual coupling is direction-dependent, leading to performance degradation of the existing algorithms. Thus, it is of great practical significance to study the DOA estimation in the presence of direction-dependent mutual coupling. Few papers are proposed to solve this problem. Based on rank-rare theory, a method of 2D-DOA estimation for direction-dependent MC is proposed in [14]. However, in order to estimate the direction-dependent MC coefficients, the algorithm adopts the idea of receiving mutual-impedance method proposed in [15], which is an off-line measurement algorithm. Therefore, the algorithm fails in the time-varying systems. In [16] Ahmet proposed a method to calibrate the direction-dependent mutual coupling and estimate the DOAs. The algorithm divides the angle search range into several sectors by means of angle sector. By comparing the spectral peaks in each sector, the angle interval of initial angle and the corresponding MC coefficients are estimated, and then self-calibration is completed by iterations. However, this method is easy to fail when the angular spacing between the DOAs of the incident signals is small or the initial value deviation is large.

Motivated by these facts, in this paper a new algorithm for estimation of DOAs and MC coefficients is proposed based on the idea of time-frequency distributions (TFDs) which has been widely applied in blind source separation [17,18]. Firstly, the space-time-frequency distributions (STFDs) matrix of the received signal is solved. Then, the single-source time-frequency (TF) points are extracted by denoising and removing the cross-terms at each TF point, and the optimal STFDs matrix of each signal is estimated by clustering the single-source TF points. Finally, the STFDs matrix is decomposed into eigenvectors and noise subspaces of each signal, and an alternating iteration method based on least squares is proposed for estimation of DOA and MC coefficients. The simulation results show that the algorithm can provide satisfactory performance in case of direction-dependent MC. The main contributions are as follows:

(1) Time-frequency analysis is utilized to solve the problem of direction-dependent mutual coupling in proposed approach.
(2) Compared with the existing algorithms, the proposed method is improved in estimation accuracy and robustness against mutual coupling.
(3) The proposed method can achieve DOA estimation under multipath or underdetermined conditions.

The rest of the paper is organized as follows: Section 2.1 is devoted to the problem formulation. Then an approach based on the TFDs is proposed to obtain the steering vector and noise subspace of each signal by separating the mixed signals, and DOAs and MC coefficients are estimated by a two-step alternate iteration in Section 2.2. Next the algorithm analysis is provided in Section 2.3. Simulations are conducted to illustrate the effectiveness of the proposed methods in Section 3 and conclusions are finally drawn in Section 4.

## 2. Models and Methods

### 2.1. Array Signal Model

Considering *K* far-field narrow-band signals $s_{k}(t)(k=1,2,\cdots ,K)$ impinging on the array which is composed of *M* elements, and the directions of arrival are $\left\{\theta_{1},\theta_{2},\cdots ,\theta_{K}\right\}$, respectively. Then the received signals at the *t*-th sample can be expressed as:

(1) X(t)=A(θ)s(t)+N(t)t=1,2,⋯,T

where $X(t)={\left[x_{1}(t),x_{2}(t),\ldots ,x_{M}(t)\right]}^{T}$ are the array outputs. $N(t)={\left[n_{1}(t),n_{2}(t),\ldots ,n_{M}(t)\right]}^{T}$ denotes zero-mean additive white Gaussian noise. $A\left(\theta \right)=[a(\theta_{1}),a(\theta_{2}),\cdots ,a(\theta_{K})]$ is the ideal manifold matrix, $a(\theta_{i})$ is the steering vector of the *i*-th signal.

In the presence of direction-dependent MC, the real steering vector is written as:

(2) b(θ)=C(θ)a(θ)

where $C(\theta )\in C^{M\times M}$ is the MC matrix.

For a uniform linear array or a uniform circular array model, the MC matrix can be expressed as a band complex symmetric Toeplitz matrix or a three-band complex cyclic matrix, respectively. The following transformation form is used to represent the MC matrix uniformly:

(3) $$C(\theta_{k})=CMC\left(c(\theta_{k})\right)$$

where CMC(·) is the operation of constructing the MC matrix using MC coefficients. $c(\theta_{k})={\left[c_{1k},c_{2k},\cdots ,c_{pk},0_{M-p}\right]}^{T}\in C^{M\times 1}$ are the MC coefficients, **0***_M–p_* is a 1 × (*M–p*) zero vector and *p* is the degree of freedom of MC, which equals the number of non-zero elements of $c(\theta_{k})$, so in the calculation process, only the first *p* elements are solved. Thus, the simplified vector ${\left[c_{1k},c_{2k},\cdots ,c_{pk}\right]}^{T}$ is used in the formula derivation and simulation conditions. Taking the uniform linear array as an example, the MC matrix of $\theta_{k}$ can be expressed as:

(4) $$C\left(\theta_{k}\right)={\left[\begin{array}{ccccc} c_{1k} & \cdots & c_{pk} & & \\ \vdots & \ddots & & \ddots & \\ c_{pk} & & c_{1k} & & c_{pk}\\ & \ddots & & \ddots & \vdots \\ & & c_{pk} & \cdots & c_{1k} \end{array}\right]}_{M\times M}$$

Then the array receiving model is expressed as

(5) X(t)=B(θ)s(t)+N(t)

where $B(\theta )=[b(\theta_{1}),b(\theta_{2}),\cdots ,b(\theta_{K})]=[C(\theta_{1})a(\theta_{1}),C(\theta_{2})a(\theta_{2}),\cdots ,C(\theta_{K})a(\theta_{K})]$ is the real array manifold matrix containing direction-dependent MC.

### 2.2. Method of DOA and MC Coefficients Estimation

**Figure 1:**
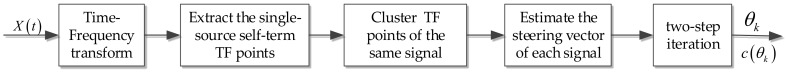
The flow diagram of the proposed algorithm.

In this section, the TFDs are introduced to obtain the true steering vector and noise-subspace of each signal, with which DOA and MC coefficients are estimated. The algorithm flow is shown in Figure 1.

The first step is obtaining the STFD matrix by quadric time-frequency transform of ***X***(*t*). Secondly the single-source self-term TF points are extracted by denoising and removing the cross-term and common-term generated by the signals. After Step 2, the optional STFD matrix of each signal is obtained by clustering TF points of the same signal. Then the steering vector and noise-subspace of each signal are estimated by the eigen-decomposition of STFD matrix. Finally, the two-step iteration is performed to estimate the DOAs and corresponding MC coefficients. The following is the method of DOA and MC coefficient estimation. Firstly, the basic concept of the TFDs and the steps of steering vector estimation are introduced.

#### 2.2.1. Steering Vector Estimation Based on TFDs

For a single signal *x*(*t*), the TFDs in discrete time form can be expressed as:

(6) $$\rho_{xx}(t,f)=\sum_{k=-\infty }^{+\infty }\sum_{l=-\infty }^{+\infty }x(t+k+l)x^{*}(t+k-l)\varphi (k,l)e^{-j4\pi fl}$$

where φ(k,l) is the kernel function, ${\left(\cdot \right)}^{*}$ denotes the conjugate operator, $\rho_{xx}(t,f)$ is the self-term TF point of x(t). 

For two signals *x*_1_(*t*) and *x*_2_(*t*), the cross-time-frequency distributions in discrete time form can be expressed as:

(7) $$\rho_{x_{1}x_{2}}(t,f)=\sum_{k=-\infty }^{+\infty }\sum_{l=-\infty }^{+\infty }x_{1}(t+k+l)x_{2}^{*}(t+k-l)\varphi (k,l)e^{-j4\pi fl}$$

$\rho_{x_{1}x_{2}}(t,f)$ is the cross-term TF point of *x*_1_(*t*) and *x*_2_(*t*).

Therefore, the STFDs matrix of ***X***(*t*) is defined as

(8) $$D_{XX}(t,f)=\sum_{k=-\infty }^{+\infty }\sum_{l=-\infty }^{+\infty }X(t+k+l)X^{H}(t+k-l)\varphi (k,l)e^{-j4\pi fl}$$

According to the definition in paper [17], TF points in time-frequency domains can be divided into three classes including self-term TF points, single-source self-term TF points and cross-term TF points which are expressed as $\left(t_{a},f_{a}\right)$,$\left(t_{as},f_{as}\right)$ and $\left(t_{c},f_{c}\right)$, respectively. 

The energy of self-term TF points is generated by one or more sources whose cross-term energy is approximately zero. Single-source self-term TF points are generated by only the self-term of single signal, and the cross-term TF points are generated by the cross-term of the signals whose self-term energy is nearly zero. 

Under the noiseless condition, substituting Equation (5) into Equation (8), we obtain:

(9) $$\begin{array}{ll} D_{XX}(t,f) & =\sum_{k=-\infty }^{+\infty }\sum_{l=-\infty }^{+\infty }Bs(t+k+l)s^{H}(t+k-l)f(k,l)e^{-j4\pi fl}B^{H}\\ & =B(\sum_{k=-\infty }^{+\infty }\sum_{l=-\infty }^{+\infty }S(t+k+l)S^{H}(t+k-l)f(k,l)e^{-j4\pi fl})B^{H}\\ & =BD_{ss}(t,f)B^{H} \end{array}$$

where $D_{XX}$ is the STFDs matrix of array outputs. $D_{ss}$ is the STFDs matrix of the incident signals whose principal diagonal elements are generated by the self-term of incident signals and the non-principal diagonal elements are corresponding to the cross-terms between the signals.

For the self-term TF points, since the signal energy is mainly generated by the self-term of sources, the non-diagonal element of $D_{ss}$ is approximately zero, so it can be expressed as a diagonal matrix, so the STFDs matrix can be expressed as:

(10) $$D_{XX}(t_{a},f_{a})=B[\begin{array}{cccc} \rho_{s_{1}s_{1}}(t_{a},f_{a}) & 0 & \ldots & 0\\ 0 & \rho_{s_{2}s_{2}}(t_{a},f_{a}) & & \vdots \\ \vdots & & \ddots & 0\\ 0 & \ldots & 0 & \rho_{s_{K}s_{K}}(t_{a},f_{a}) \end{array}]B^{H}$$

On this basis, when the energy at the TF points is generated only by the single source $s_{i}\;\;(i=1,2\cdots K)$, $D_{XX}(t_{as},f_{as})\;$ can be expressed as:

(11) $$D_{XX}(t_{as},f_{as})\;=\rho_{s_{i}s_{i}}(t_{as},f_{as})a_{i}a_{i}^{H}$$

It is deduced from Equation (11) that the steering vector of each signal can be obtained by eigen-decomposition of the STFDs matrix at the TF points of single-source self-term. The procedure of extracting TF points from single source term is as follows.

*Step 1*:  Remove the noise: In order to reduce the computation burden and improve the estimation accuracy, it is necessary to set an appropriate threshold to remove the TF points with low energy which may be generated by noise. For each time slice $\left(t_{p},f\right)$, apply Equation (12) for all frequency $f_{q}$ points in this slice, and then the TF points with large energy remain:
  (12) $$\frac{\left\|D_{XX}(t_{p},f_{q})\right\|}{\underset{f}{\max}\{\left\|D_{XX}(t_{p},f)\right\|\}}>\varepsilon_{1}$$
  where $\varepsilon_{1}$ is a small positive real number and ‖•‖ represents the operator of 2-norm.
*Step 2*:  Extract the TF points of self-term: After removing the noise points, the retaining TF points mainly include self-term TF points and cross-term TF points. At the self-term TF points, the STFDs matrix is approximately diagonal, and the values of the principal diagonal elements are much larger than those of the other elements, so the STFDs matrix at self-term TF points yields:
  (13) $$\frac{trace\{D_{XX}(t,f)\}}{\left\|D_{XX}(t,f)\right\|}>\varepsilon_{2}$$
  where $\varepsilon_{2}$ is a positive real number close to but less than 1.
*Step 3*:  Extract the TF points of single-source self-term: For signals which overlap in time-frequency domain, the self-term TF points may be composed of multiple signals. Therefore, it is necessary to extract the single-source self-term TF points from the self-term TF points, which can be accomplished by Equation (14):
  (14) $$\left|\frac{\lambda_{\max}\{D_{XX}(t,f)\}}{trace\{D_{XX}(t,f)\}}-1\right|\leq \varepsilon_{3}$$
  where $\varepsilon_{3}$ is a small positive real threshold and $\lambda_{\max}\{D_{XX}(t,f)\}$ is the largest eigenvalue of $D_{XX}(t,f)$.
  With the three steps above, the single-source self-term TF points are obtained. Then the steering vectors of each signal as well as the noise subspace could be estimated by the eigen-decomposition of their STFDs matrix. However, the above derivation is completed without considering the noise. In the presence of noise, the steering vectors estimated by only a few TF points are biased. Therefore, it is necessary to obtain more accurate information by clustering the multiple single-source self-term TF points of the same signal. A time-frequency clustering method is provided in Step 4.
*Step 4*:  Cluster the TF points of the same signal: The steering vector of each signal can be estimated as the principal eigenvector of STFDs matrix at each TF point. Regarding the steering vector which contains the DOA information as a feature, all self-term TF points can be classified into *Q*(*Q≥K*) categories by the classification algorithm. That is to say, if the following conditions are satisfied, TF points of $(t_{1},f_{1}),(t_{2},f_{2})\;$ belong to the same category:
  (15) $$d(a(t_{1},f_{1}),a(t_{2},f_{2}))<\varepsilon_{4}$$
  where *d*(***x,y***) is the Euclidean distance between ***x*** and ***y***, and $\varepsilon_{4}$ is a small positive threshold.

After clustering, we extract the first *K* categories which contain the most TF points, and obtain the TFDs of the *K* signals, respectively. Then the STFDs matrices at those TF points belonging to the first *K* categories are summed and averaged. Finally, the eigen-decomposition of the average STFDs matrix is performed to estimate the steering vector of each signal and the corresponding noise subspace, which are denoted as $\tilde{b}(\theta_{k})$ and $E_{n}(\theta_{k})$, respectively, k=1,2,⋯,K.

The above steps of removing the noise, extracting self-term TF points, and clustering TF points of same signal aimed to essentially select the TF points that satisfy the specific conditions, and then the matrix at the TF point is processed.

#### 2.2.2. DOA and MC Coefficients Estimation

For a uniform linear array or uniform circular array model, the transformation relationship between the MC coefficients vector and MC matrix can be expressed by Equation (16):

(16) $$b\left(\theta_{k}\right)=C\left(\theta_{k}\right)a\left(\theta_{k}\right)=T\left(\theta_{k}\right)c\left(\theta_{k}\right)\;k=1,2,\cdots ,K$$

where $T(\theta_{k})$ is transformation matrix which contains the direction information.

For a uniform linear array, the transformation matrix can be expressed as:

(17) $$T\left(\theta_{k}\right)=Q_{1}\left(\theta_{k}\right)+Q_{2}\left(\theta_{k}\right)$$

where:

(18) $$\begin{array}{l} {\left[Q_{1}\left(\theta_{k}\right)\right]}_{ij}=\left\{ \begin{array}{ll} {\left[a\left(\theta_{k}\right)\right]}_{i+j-1} & i+j\leq M+1\\ 0 & i+j>M+1 \end{array}\right.\\ {\left[Q_{2}\left(\theta_{k}\right)\right]}_{ij}=\left\{ \begin{array}{ll} {\left[a\left(\theta_{k}\right)\right]}_{i-j+1} & i\geq j\geq 2\\ 0 & otherwise \end{array}\right. \end{array}$$

Similarly, for a uniform circular array the transformation matrix can be expressed as:

(19) $$T\left(\theta_{k}\right)=Q_{1}\left(\theta_{k}\right)+Q_{2}\left(\theta_{k}\right)+Q_{3}\left(\theta_{k}\right)+Q_{4}\left(\theta_{k}\right)$$

where $Q_{1}(\theta_{k})$, $Q_{2}(\theta_{k})$, $Q_{3}(\theta_{k})$, $Q_{4}(\theta_{k})$ yield:

(20) $$\begin{array}{l} {\left[Q_{1}\left(\theta_{k}\right)\right]}_{ij}=\left\{ \begin{array}{ll} {\left[a\left(\theta_{k}\right)\right]}_{i+j-1} & i+j\leq M+1\\ 0 & i+j>M+1 \end{array}\right.\\ {\left[Q_{2}\left(\theta_{k}\right)\right]}_{ij}=\left\{ \begin{array}{ll} {\left[a\left(\theta_{k}\right)\right]}_{i-j+1} & i\geq j\geq 2\\ 0 & otherwise \end{array}\right.\\ {\left[Q_{3}\left(\theta_{k}\right)\right]}_{ij}=\left\{ \begin{array}{ll} {\left[a\left(\theta_{i}\right)\right]}_{M+1+i-j} & i<j\leq l\\ 0 & otherwise \end{array}\right.\\ {\left[Q_{4}\left(\theta_{k}\right)\right]}_{ij}=\left\{ \begin{array}{ll} {\left[a\left(\theta_{k}\right)\right]}_{i+j-M-1} & 2\leq i\leq l,i+j\geq M+2\\ 0 & otherwise \end{array}\right. \end{array}$$

As there is a multiple relation between the estimated steering vector and the actual steering vector, we deduce that:

(21) $$b\left(\theta_{k}\right)=\rho_{k}\tilde{b}\left(\theta_{k}\right)=C\left(\theta_{k}\right)a\left(\theta_{k}\right)=T\left(\theta_{k}\right)c\left(\theta_{k}\right)$$

where *ρ_k_* is the multiplier. And the MC coefficients $c(\theta_{k})$ can be calculated by the least squares method:

(22) $$\tilde{c}\left(\theta_{k}\right)=\rho_{k}{\left(T^{H}\left(\theta_{k}\right)T\left(\theta_{k}\right)\right)}^{-1}T^{H}\left(\theta_{k}\right)\tilde{b}\left(\theta_{k}\right)$$

According to Equation (22), we find that there also exists a corresponding coefficient relationship between the estimated MC coefficients $\tilde{c}(\theta_{k})$ and the true MC coefficients $c(\theta_{k})$. Because the first element of $\tilde{c}(\theta_{k})$ should be 1, the actual MC coefficients can be obtained by normalizing the first element of $\tilde{c}(\theta_{k})$ with Equation (23):

(23) $$\hat{c}(\theta_{k})=\frac{\tilde{c}(\theta_{k})}{{\left[\tilde{c}(\theta_{k})\right]}_{1}}$$

where ${\left[\tilde{c}\left(\theta_{k}\right)\right]}_{1}$ represents the first element of $\tilde{c}(\theta_{k})$.

Then DOA of the *k*-th signal is estimated according to Equation (24) (root MUSIC algorithm could be applied to solve the functions) with the new MC matrix $\hat{C}(\theta_{k})=\mathrm{CMC}\left(\hat{c}(\theta_{k})\right)$:

(24) $$\underset{\theta_{k}}{\min}{\left\|E_{n}^{H}\left(\theta_{k}\right)C\left(\theta_{k}\right)a\left(\theta_{k}\right)\right\|}^{2}\;k=1,2,\cdots ,K$$

However, as the direction $\theta_{k}$ and the MC coefficients are unknown, the function cannot be solved directly. Based on the above steps, a new method for DOA estimation and MC coefficients is proposed based on the two-step alternating iteration.

Firstly, initialize the MC coefficients and compute the DOAs ${\hat{\theta }}_{1},{\hat{\theta }}_{2},\cdots ,{\hat{\theta }}_{K}$ through the conventional algorithms. Then construct the transformation matrix $T({\hat{\theta }}_{1}),T({\hat{\theta }}_{2}),\cdots ,T({\hat{\theta }}_{K})$ according to ${\hat{\theta }}_{1},{\hat{\theta }}_{2},\cdots ,{\hat{\theta }}_{K}$. On this basis, the MC coefficients is estimated by Equations (22) and (23) with which the new MC matrix is constructed. Finally the DOA of each signal is obtained using Equation (24), and transformation matrix with the new angle information is constructed. In this way, iterations are carried out alternately until the estimation bias is less than the threshold or the iterations have been repeated for certain times. The proposed algorithm is summarized in Table 1.

### 2.3. Algorithmic Analysis

1) For the selection of the TFD kernel function, it is known that different TFD kernel functions correspond to different TFD transforms, which can be divided into linear time-frequency transforms and quadratic time-frequency transforms. The Wigner-Ville distribution (WVD) is one of the quadratic TFDs that has better performance in TF focusing and resolution. These two elements are significant in the extraction and clustering of TF points. However, due to the interaction between different signals, cross-terms are generated, which cause false time-frequency information and degrade the estimation accuracy. Therefore, the smoothed pseudo-Wigner-Ville distribution (SPWVD) is utilized to suppress the cross-terms between signals by windowing method in this paper. Similarly, the short-time Fourier transform (STFT) can also provide the accurate time-frequency distribution to solve the problem and we should choose the kernel function flexibly for the different conditions.
2) This algorithm aims at the estimation of direction-dependent MC, but it is also applicable in the presence of a single MC. The single MC coefficients can be obtained by averaging the estimated MC coefficients of different directions.
3) The algorithm is based on the condition that the TFDs of the signals do not completely overlap, otherwise the blind separation will not be effective. This condition is easy to be obtained in practice. Even the coherent signals (co-frequency interference or multipath signals with different arrival time due to different propagation paths) has different TFDs. Therefore, the proposed algorithm is also effective for coherent signals with different arrival times.
4) This algorithm is able to estimate DOA and MC coefficients under undetermined conditions. That means, the algorithm is still effective when the number of array elements is less than the number of signals. This is because the steering vectors of each signal can be estimated using TFDs, which is similar to estimating the DOA and MC coefficients of each signal separately.
5) For the selection of empirical parameters, we find that $\varepsilon_{1}$ is the threshold for noise points removing which depends on the ratio between the power of noise and signals. The lager the ratio is, the larger $\varepsilon_{1}$ should be. However, the power of noise distributes in the whole TF domain uniformly while the power of signals mainly distributes on a few TF points. Therefore, the noise to signal ratio is small in general. Usually we set $\varepsilon_{1}\leq 0.3$ in this paper. $\varepsilon_{2}$ is the threshold for the self-term TF points extraction which approaches to 1 and $\varepsilon_{2}=0.9$ in this paper. $\varepsilon_{3}$ is a small positive threshold for the single-source TF points extraction and is fixed at 0.2 in this paper. $\varepsilon_{4}$ is set to cluster the TF points into Q categories and we set $\varepsilon_{4}=0.2$.

## 3. Numerical Simulation

This section illustrates the effectiveness of the algorithm through simulations with other methods as comparison. Without loss of generality, a uniform linear array composed of 7 elements is selected in this paper, and p=3. Assuming that there are three far-field narrowband LFM (linear frequency modulation) signals in the space, and the duration of three signals is the same. The sampling frequency of system is 50 MHz. The parameters of signals are listed in Table 2. 

### 3.1. Calibration Results of Proposed Algorithm

**Figure 2:**
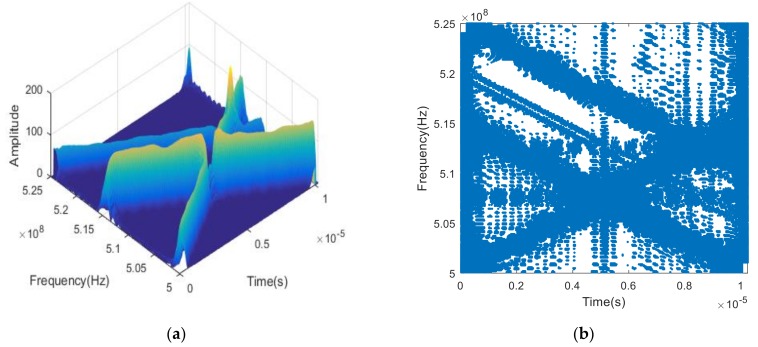
Time-Frequency Distribution of received signals. (**a**) Time-Frequency Distribution (3-dimensional); (**b**) Time-Frequency Distribution (2-dimensional).

In this section, we provide the results of each step shown in Section 3.1 to verify the effectiveness of the algorithm. Figure 2a shows a three-dimensional time-frequency diagram of the received signal. From which, we find that cross-terms exist in the time-frequency domain of the three signals. Although the smooth pseudo-Wigner distribution is used to remove most of the cross-terms, there still remain some cross-terms in the overlapping part. Figure 2b is a two-dimensional time-frequency distribution which has been binarily processed. It can be seen that except for the time-frequency points of the signal itself, there are also noise points and the time-frequency points of cross-terms between different signals.

**Figure 3:**
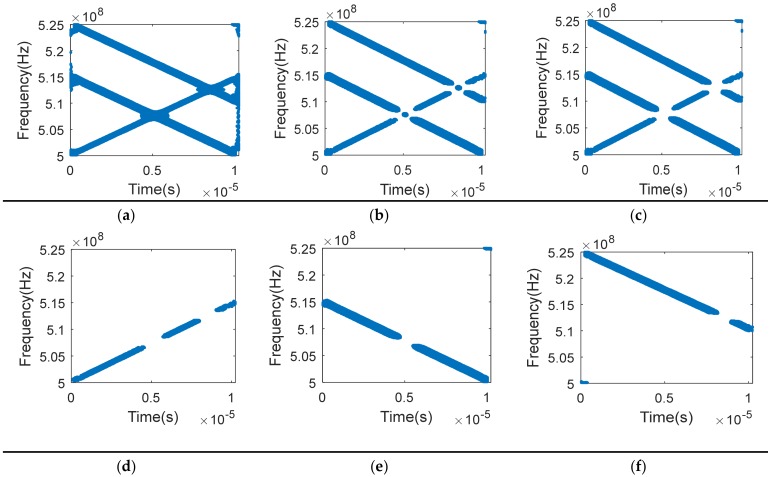
Processing steps of TFDs. (**a**)The TFDs after removing noise points; (**b**) The TFDs of self-term points; (**c**) The TFDs of single-source self-term points; (**d**)The TFDs of signal 1 after clustering; (**e**) The TFDs of signal 2 after clustering; (**f**) The TFDs of signal 3 after clustering.

Figure 3 indicates the processing results introduced in Section 3.1. Figure 3a shows the result of removing the noise points, in which we find that nearly all the noise points can be filtered, while only the TF points with high energy remain. Figure 3b shows the result of the self-term TF points extraction. It is seen that the cross-term TF points are removed but the self-term TF points in the overlapping area are removed as well. Figure 3c shows the result of the single-source self-term TF points extraction. We find that there are only single-source TF points remaining while the overlapping self-term TF points of multiple signals are removed. Figure 3d–f are the TF distribution diagrams of Signals 1–3 after clustering, respectively. It is found that the TF points of each signal can be obtained by clustering with spatial information as feature. And they are matched with the modulation frequency of the real signals.

**Figure 4:**
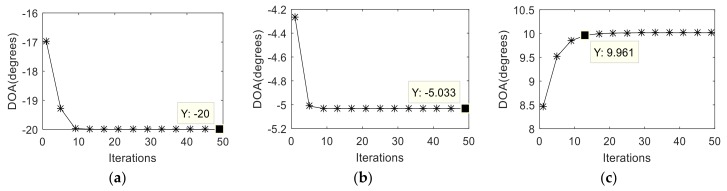
Variation of the estimated DOAs versus the iterations of three signals. (**a**) Variation of the estimated DOAs versus the iterations of signal 1; (**b**) Variation of the estimated DOAs versus the iterations of signal 2;(**c**) Variation of the estimated DOAs versus the iterations of signal 3.

**Figure 5:**
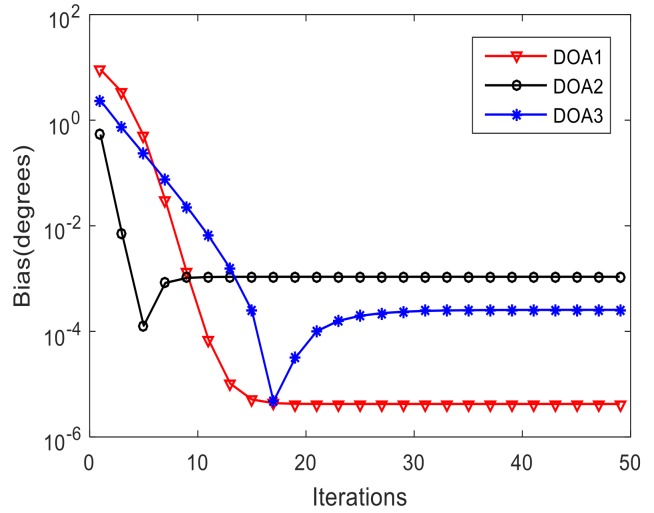
Variation of the estimated DOAs versus the iteration times.

**Figure 6:**
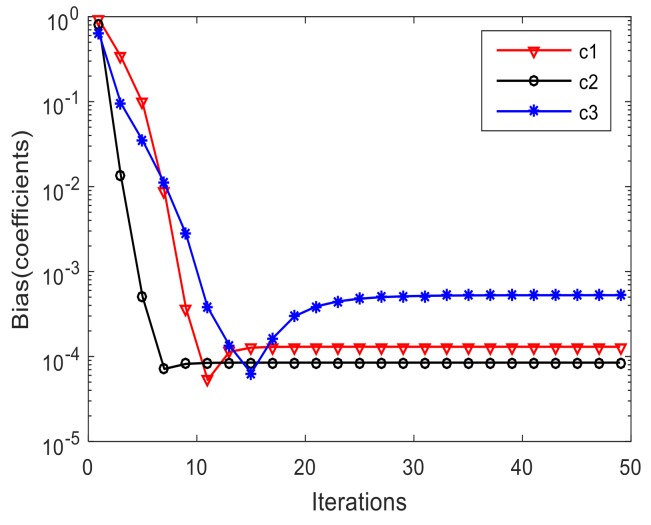
Variation of the estimated MC coefficients versus the iteration times.

Figure 4a–c show the variation of the estimated DOAs versus the iteration times of three signals respectively. We find that after a few times of iteration, the estimated DOAs gradually approach the real value and finally stabilize. Figure 5 and Figure 6 indicate variation of the estimated DOAs and MC coefficients versus the iteration times respectively. It can be seen from the figures that the algorithm has high estimation accuracy in DOA and MC coefficients under the condition of SNR=10 dB after a few times of iteration.

### 3.2. RMSE Comparison versus Input Signal-Noise-Ratio (SNR)

In this section, performance of the proposed algorithm is investigated with different input SNR from −10 dB to 14 dB, which is compared with PEDDMC algorithm [16], conventional MUSIC and the Cramer-Rao lower bound (CRLB) with unknown mutual coupling [7]. The RMSE are obtained through 200 Monte-Carlo simulations. The calculation formula of RMSE is as follows:

(25) $$RMSE=\sqrt{\sum_{n=1}^{N_{s}}\sum_{i=1}^{K}{\left(\theta_{i}-{\hat{\theta }}_{i,n}\right)}^{2}/\left(KN_{s}\right)}$$

**Figure 7:**
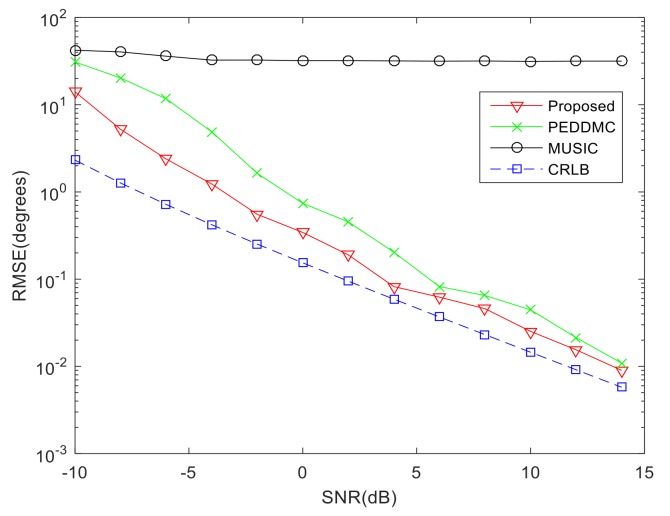
Comparison of DOA estimation performance versus SNR.

As shown in the Figure 7, when the SNR is low, estimation errors of the three algorithms are large. With the increase of SNR, performance of the proposed algorithm and PEDDMC algorithm are improved. However, the proposed algorithm has lower RMSE because the TFDs algorithm has better anti-noise performance, and in addition the DOA estimation and error calibration for each signal are carried out separately. As a result, the proposed method has better performance and its RMSE follows the CRLB closely. As for the MUSIC algorithm, it is a classical super-resolution algorithm with superior estimation performance and robustness under ideal conditions, but it fails in the presence of mutual coupling conditions, since it has no ability to achieve calibration. As a result its performance does not improve as SNR increases.

### 3.3. RMSE Comparison versus Input Snapshots

**Figure 8:**
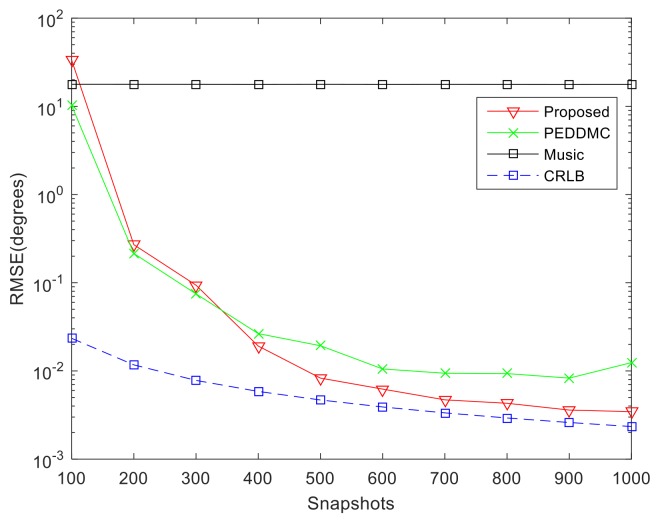
Comparison of DOA estimation performance versus snapshots.

This section compares the performance of the algorithms with different number of snapshots from 100 to 1000. We fix the SNR at 15 dB and calculate the output RMSE through 200 Monte Carlo simulations. From Figure 8, it is known that the estimation performance of proposed algorithm is poor under the condition of small snapshots. This is because small snapshots will lead to the TF resolution deterioration of time-frequency distribution, which affects the extracting and clustering of TF points from single-source self-term. With the increase of snapshots, the RMSE of the proposed algorithm gradually becomes lower than that of the PEDDMC algorithm and becomes very close to the CRLB.

## 4. Conclusions

Considering the problem that the performance of traditional calibration algorithms degrades in the presence of direction-dependent mutual coupling, this paper introduces the time-frequency analysis method into array signal processing and proposes a new self-calibration algorithm based on alternating iteration. The simulation results show that the proposed algorithm is effective in the presence of direction-dependent mutual coupling and outperforms the existing algorithms. Though the computational complexity of the proposed algorithm is higher because it requires more snapshots to ensure the estimation accuracy of time-frequency transform, compared with the other existing algorithms, the proposed algorithm can perform DOA estimation under multipath or underdetermined conditions. 

## 5. Patents

The authors would like to thank the editor and anonymous reviewers for their careful reading and constructive comments which provide an important guidance for our paper writing and research work. This work was supported by the National Natural Science Foundation of China under Grant 61703433.

## Figures and Tables

**Table 1:** Algorithm for estimation of DOA and direction-dependent MC based on TFDs.

Step 1. Collect N snapshots and calculate the STFDs of received signal with (9).
Step 2. Extract the single source TF points based on (12)–(14).
Step 3. Cluster the single source TF points into *Q*(*Q≥K*) categories using (15).
Step 4. Estimate $\tilde{b}(\theta_{k})$ and $E_{n}(\theta_{k})$ of each signal with the ***K*** largest classes.
Step 5. Construct the transformation matrix with $\tilde{b}(\theta_{k})$.
Step 6. Estimate the MC coefficients based on Equations (22) and (23).
Step 7. Construct the MC matrix using the new MC coefficients and estimate the DOAs using (24).
Step 8. Repeat Step 5 to Step 7 until the estimation errors is less than the threshold or the iterations have been repeated for certain times.

**Table 2:** Parameters of Linear Frequency Modulation Signals.

Parameters	LFM Signal 1	LFM Signal 2	LFM Signal 3
Frequency	500 MHz–515 MHz	515 MHz–500 MHz	525 MHz–510 MHz
DOA	$-20^{o}$	$-5^{o}$	$10^{o}$
Snapshots	512	512	512
SNR	10 dB	10 dB	10 dB
MC coefficients	$\left[\begin{array}{c} 1\;\\ 0.623+i0.587\;\\ 0.365+i0.241 \end{array}\right]$	$\left[\begin{array}{c} 1\;\\ 0.579+i0.502\;\\ 0.314+i0.321 \end{array}\right]$	$\left[\begin{array}{c} 1\;\\ 0.427+i0.604\;\\ 0.256+i0.336 \end{array}\right]$
